# New concepts in diabetic kidney disease

**Published:** 2015-01-12

**Authors:** Parisa Motamedi, Nahid Dehghani, Fereshte Kiani, Sara Torkamaneh, Hamid Nasri

**Affiliations:** ^1^Environmental Health Engineering, Engineering Department, Health Faculty, Isfahan University of Medical Sciences, Isfahan, Iran; ^2^Young Researchers and Elite Club, Isfahan (Khorasgan) Branch, Islamic Azad University, Isfahan, Iran; ^3^Department of Biostatistics and Epidemiology, School of Health, Isfahan University of Medical Sciences, Isfahan, Iran; ^4^Department of Physical Education and Sport Sciences, Khorasgan University of Isfahan, Isfahan, Iran; ^5^Department of Nephrology, Isfahan University of Medical Sciences, Isfahan, Iran

**Keywords:** Diabetic nephropathy, Medicinal plants, Kidney

**Implication for health policy/practice/research/medical education:**

Diabetic nephropathy refers to any deleterious effect on kidney structure and/or function due to diabetes mellitus. One of the most severe and common complications of diabetes is renal disease. There are lots of other medicinal plants, with antioxidant and hypoglycemic activities. Most of the plants with antioxidant activities have been shown to reduce diabetic nephropathy too. Whether or not these plants act with the same mechanism is not clear and worth examining.

Diabetic nephropathy refers to any deleterious effect on kidney structure and/or function due to diabetes mellitus. One of the most severe and common complications of diabetes is renal disease. The majority of patients with 18 years’ duration show the signs of diabetic renal involvement (1-3). About one third of patients with type 1 or 2 diabetes mellitus develop end stage renal disease which proceeds to diabetic nephropathy, the principal cause of mortality and morbidity in diabetic patients. The onset of diabetic nephropathy is associated with a progressive rate of urinary albumin excretion and glomerular filtration rate, and then decline in renal function (1-5). One of the main causes of change in kidney function of diabetic patients is sustained hyperglycemia. Although the exact mechanism in this effect is not fully established, however, hyperglycemia enhances oxidative stress, increase in formation of advanced glycation end-products and activation of hexosamine flux, causing inflammation and renal damage (2-6). Advanced glycation end-products reduce matrix protein flexibility through cross-link formation of the extracellular matrix proteins. This may lead to abnormal interactions with other matrix components. Furthermore, increase in production of advanced glycation end-products usually results in an increase in production of proteins of mesangial cells and macrophages extracellular matrix as well as in endothelial cells, in the kidney. The mesangial alteration is the main cause of decrease in renal function of diabetic patients (1-7). As nephropathy progresses, the decline in glomerular filtration rate may be due to expansion of the mesangial matrix. This phenomenon compresses the glomerular capillaries, resulting in reduction in the filtration surface area and impairment in maintaining the normal glomerular capillary hydrostatic pressure (4). The reduction in glomerular filtration rate also causes reduction in sodium load delivery to the macula densa cells, resulting in an increase in tubulo-glomerular feedback (3-9). Hence, due to hyperactivation of the renin–angiotensin–aldosterone system, the production of angiotensin II increases resulting in more reabsorption of sodium. This, in turn, increases the systemic blood pressure (2-8). Blocking the advanced glycation end-products receptor or inhibition of advanced glycation end-products formation causes reno-protection in diabetic animals (4-10). The accumulation of advanced glycation end-products, which is the main cause of mesangial alteration and decrease in renal function in diabetic patients, can be prevented by antioxidants (3-9). Metformin, a biguanide derivative, which is the only example of an approved anti-diabetic from a herbal source, *Galega officinalis* (French lilac) has been shown to be useful in the prevention of the development of advanced glycation end-products. There are several other antioxidant plants such as *Panax quinquefolium, Vitis vinifer*a, curcumin from *Curcuma longa* and glycosides from *Stelechocarpus caulifloru* have also been reported to inhibit formation of advanced glycation end-products or block its receptors (6-14). There are lots of other medicinal plants such as *Cucurbita pepo, Cornus mas, turnip,* and *Prangos ferulacea,* with antioxidant and hypoglycemic activities. Most of the plants with antioxidant activities have been shown to reduce diabetic nephropathy (13-16). Whether or not these plants act with the same mechanism is not clear and worth examining.

## Authors’ contribution

All authors wrote the manuscript equally.

## Conflicts of interest

The authors declared no competing interests.

## Ethical considerations

Ethical issues (including plagiarism, misconduct, data fabrication, falsification, double publication or submission, redundancy) have been completely observed by the authors.

## Funding/Support

None.
